# Probiotic Characteristics and Anti-Inflammatory Effects of *Limosilactobacillus fermentum* 664 Isolated from Chinese Fermented Pickles

**DOI:** 10.3390/antiox13060703

**Published:** 2024-06-07

**Authors:** Huichao Hao, Ziyu Nie, Yanyang Wu, Zhiwei Liu, Fenglian Luo, Fangming Deng, Lingyan Zhao

**Affiliations:** College of Food Science and Technology, Hunan Agricultural University, Changsha 410128, China; 13117336402@163.com (H.H.); 13467663507@163.com (Z.N.); wuyanyang2002@126.com (Y.W.);

**Keywords:** lactic acid bacteria, probiotic, pickle, anti-inflammation, oxidative stress

## Abstract

*Limosilactobacillus fermentum* (*L. fermentum*) is widely used in industrial food fermentations, and its probiotic and health-promoting roles attracted much attention in the past decades. In this work, the probiotic potential of *L. fermentum* 664 isolated from Chinese fermented pickles was assessed. In addition, the anti-inflammatory properties and mechanisms were investigated using lipopolysaccharide (LPS)-stimulated RAW264.7 cells. Results indicated that *L. fermentum* 664 demonstrated excellent acid and bile salt tolerance, adhesion capability, antimicrobial activity, and safety profile. *L. fermentum* 664 downregulated the release of inflammatory mediators, including tumor necrosis factor-α (TNF-α), interleukin-6 (IL-6), interleukin-1β (IL-1β), and cyclooxygenase-2 (COX-2) stimulated with LPS. Moreover, *L fermentum* 664 inhibited the nuclear translocation of the nuclear factor κB (NF-κB) and the activation of mitogen-activated protein kinases (MAPKs) induced by LPS. This action was associated with a reduction in reactive oxygen species (ROS) levels and an enhanced expression of heme oxygenase-1 (HO-1) protein. Additionally, whole genome sequencing indicated that *L. fermentum* 664 contained genes that encode proteins with antioxidant and anti-inflammatory functions, including Cytochrome bd ubiquinol oxidase subunit I (CydA), Cytochrome bd ubiquinol oxidase subunit II (CydB), and NAD(P)H dehydrogenase quinone 1 (NQO1). In conclusion, our study suggested that *L. fermentum* 664 has the potential to become a probiotic and might be a promising strategy for the prevention of inflammation.

## 1. Introduction

Probiotics are dietary supplements or food ingredients that promote a healthy balance of microorganisms in the gastrointestinal tract, resulting in various beneficial effects on the body [1]. Lactic acid bacteria (LAB), such as *Lactobacillus acidophilus* [2], *Lactobacillus rhamnosus* GG (LGG) [3], *Lactobacillus fermentum* [4], and *Lactiplantibacillus plantarum* (*L. plantarum*) [1], are among the most frequently utilized microorganisms in probiotic applications. These probiotic bacteria are commonly derived from the gastrointestinal tract of animals or humans and have a positive influence on the host by modulating the gut microbiota, such as inhibiting the adhesion of enteropathogenic bacteria [5], preventing diarrhea [6], and relieving inflammation [7]. Currently, there is a growing interest in unconventional sources such as fermented foods. It has been discovered that strains derived from such sources exhibit good resistance to high acidity, bile salts, gastric proteases, and pancreatic proteases. These strains can maintain their viability in the human gastrointestinal tract and provide benefits to the host through their own metabolic activities. Manoramade et al. [8] screened an anti-inflammatory bacterium *L. fermentum* NCDC400 from fermented dairy products, which was shown to downregulate pro-inflammatory cytokines tumor necrosis factor-α (TNF-α), interleukin-6 (IL-6), interleukin-1β (IL-1β), and nitric oxide (NO) in lipopolysaccharide (LPS)-stimulated RAW264.7 cells by secreting exopolysaccharides (EPS). *L. fermentum* MF10, which was isolated from Korean Kimchi, protected HT-29 and HaCaT cells from LPS damage by modulating pro-inflammatory cytokines, oxidative biomarkers, and membrane potential [9]. Pickles, which are traditional Chinese fermented vegetables, not only hold significant cultural value but also contribute to the development of the regional economy. Studies have shown that fermented pickles can provide physiological functions, including preventing fat accumulation, maintaining redox homeostasis, and relieving inflammation [10]. Kai et al. [11] revealed that Sichuan pickles from China could significantly control obesity induced by a high-fat diet in mice, thereby reducing the risk of lipid metabolic disorders. Additionally, pickles were found to contain antioxidant and antimicrobial phenolic compounds, such as 2,6-dihydroxyacetophenone, 4-hydroxybenzaldehyde, and 4-hydroxyphenethyl alcohol [12]. These beneficial effects are closely related to the microorganisms grown in them [13,14]. However, the effect of probiotics derived from pickles on host health has been poorly investigated.

Inflammation is a necessary part of the body in response to chemical irritation, microbial infection, and physical damage. If inflammation is not controlled, it will cause tissue damage and further contribute to the development of chronic pathologies such as inflammatory bowel disease, cardiovascular disease, and diabetes [15,16]. When the body is exposed to external stimuli, the pro-inflammatory mediators, including reactive oxygen species (ROS), cyclooxygenase-2 (COX-2), and pro-inflammatory cytokines secreted by immune cells, play a crucial role in transmitting inflammatory signals [17]. Nuclear translocation of the nuclear factor κB (NF-κB) and the activation of mitogen-activated protein kinases (MAPKs) family members, including extracellular signal-regulated kinase (ERK), c-Jun N-terminal kinase (JNK), and p38, have been suggested as effective targets for treating inflammation. Inhibiting their activation can reduce the expression of pro-inflammatory mediators [18,19]. According to the “hygiene hypothesis”, the diseases mediated by inflammation may be related to intestinal microbial disorders [20,21]. Due to the immunoregulatory effects in the gut, probiotics have emerged as potential agents for treating inflammation [22]. Our pre-experiments isolated 49 LAB strains from various Chinese fermented pickle products, and assessed their anti-inflammatory effects on RAW264.7 cells exposed to LPS. *L. fermentum* 664 was the strain with the highest anti-inflammatory potential.

Therefore, this study aimed to evaluate the probiotic potential and anti-inflammatory effect of *L. fermentum* 664. To investigate the fundamental characteristics of probiotics, we explored their acid resistance, tolerance to bile salts, adhesion ability, and antimicrobial activity. To assess the anti-inflammatory effects of *L. fermentum* 664, RAW264.7 cells were treated with *L. fermentum* 664 suspension at a concentration of 1 × 10^10^, 1 × 10^8^, and 1 × 10^6^ CFU/mL (the concentration selection was based on similar studies conducted previously), respectively, and then were stimulated with LPS, which increased the expression of pro-inflammatory mediators, to induce an inflammatory response. Pro-inflammatory cytokines, ROS, COX-2, heme oxygenase-1 (HO-1), NF-κB, and MAPKs were monitored as inflammation biomarkers. For the application of *L. fermentum* 664, we analyzed the antibiotic resistance, the potential anti-inflammatory substances, and the anti-inflammatory signaling pathways of *L. fermentum* 664 based on whole-genome sequencing.

## 2. Materials and Methods

### 2.1. Isolation and Preservation of Strains

These pickles were made from salt-preserved radish leaves, and they were purchased from a farmer in Chenxi County, Huaihua City, Hunan Province, China. A small number of pickles was thoroughly mixed with sterile phosphate buffered saline (PBS, 0.1 M, pH 7.2) on a clean bench. The solution was subjected to gradient dilution. A total of 0.1 mL of the diluted solution was inoculated into a modified Chalmers (MC, Guangdong Huankai Microbiology Technology Co., Ltd., Guangzhou, China) agar medium containing 0.3% CaCO_3_. After incubation for 48 h at 37 °C under anaerobic conditions, a single colony with a clear zone of calcium solubilization was transferred into 10 mL of de Man, Rogosa, and Sharpe (MRS, Guangdong Huankai Microbiology Technology Co., Ltd., Guangzhou, China) broth and anaerobically inoculated at 37 °C for 12 h. After cultivation, an equal volume of 40% glycerol was added and the mixture was stored at −80 °C in a freezer. In addition, the isolated strains were inoculated onto MRS agar slants and incubated anaerobically at 37 °C for 48 h. Then, the cultures were sent for preservation to the China General Microbiological Culture Collection Center (CGMCCC). The assigned preservation code is CGMCC No. 25918.

### 2.2. Bacterial Strain and Cultivation Conditions

LGG (ATCC 53103) was purchased from the American Type Culture Collection (ATCC). The *L. fermentum* 664 and LGG stored at −80 °C were, respectively, cultured in MRS broth at 37 °C for 12 h. *Escherichia coli* (*E. coli*, CGMCC 9181) was purchased from CGMCCC. *Staphylococcus aureus* (*S. aureus*, ATCC 6538) and *Salmonella enterica subsp. enterica* (*S. enterica*, ATCC 14028) were purchased from ATCC. *E. coli*, *S. aureus*, and *S. enterica* were, respectively, inoculated in nutrient broth (NB, Guangdong Huankai Microbiology Technology Co., Ltd., Guangzhou, China) medium at 37 °C for 12 h. The above bacteria were subcultured three times before each experiment and cultivated under anaerobic conditions.

### 2.3. Acid Resistance Test

Acid resistance was measured according to Huligere et al. [23]. After culturing the strains in MRS medium for 12 h, the *L. fermentum* 664 was harvested by centrifugation at 10,000 rpm, kept at 4 °C for 10 min, and then washed twice with PBS (0.1 M, pH 7.2). The cell density was adjusted to 1 × 10^9^ CFU/mL in PBS. The pH value of MRS broth was adjusted to 2.0 by 0.1 M HCl. Subsequently, *L. fermentum* 664 was inoculated at a 1% (*v*/*v*) concentration into the MRS broth and incubated at 37 °C. The number of *L. fermentum* 664 was counted at 0 h and 4 h by the plate counting method. The survival rate was calculated using the following formula:

$$Survival\;rate\left(\%\right)=(\mathrm{lg}A_{1}/\mathrm{lg}A_{0})\;\times \;100$$

where *A*_1_ is the number of *L. fermentum* 664 after 4 h of incubation, and *A*_0_ is the number of *L. fermentum* 664 in the culture at 0 h.

### 2.4. Bile Salt Tolerance Test

Bile salt tolerance was measured according to Hu et al. [24]. The cell density of the activated *L. fermentum* 664 was adjusted to 1 × 10^9^ CFU/mL in PBS (0.1 M, pH 7.2). *L. fermentum* 664 was inoculated at a 1% concentration into the MRS broth with 0.3% (*w*/*v*) bile salts and was incubated at 37 °C for 2 h. The number of *L. fermentum* 664 was counted at 0 h and 2 h by the plate counting method. The survival rate was calculated using the following formula:

$$\mathrm{Survival}\;\mathrm{rate}\;(\%)=(\mathrm{lg}A_{3}/\mathrm{lg}A_{2})\;\times \;100$$

where *A*_3_ is the number of *L. fermentum* 664 after 2 h of incubation. *A*_2_ is the number of *L. fermentum* 664 in the culture at 0 h.

### 2.5. Cell Surface Hydrophobicity Measurement

The cell surface hydrophobicity percentage of *L. fermentum* 664 was determined using the method mentioned by Darmastuti et al. [25]. Xylene, as a hydrophobic solvent, is commonly used to determine the hydrophobicity of bacterial strains. Its low volatility and high stability ensure experimental reproducibility [26]. The activated *L. fermentum* 664 was washed twice and then resuspended in PBS (0.1 M, pH 7.2) to a final optical density at 600 nm (OD_600_) of 0.5–0.6 (*B*_0_). The suspension was added with xylene in a ratio of 3:1 (*v*/*v*), and the mixture was fully shaken for 1 min. The resulting solution was incubated for 10 min at room temperature and then vortexed for 2 min. Then, the suspension was kept undisturbed at 37 °C for 20 min, and the final OD_600_ (*B*_1_) of the aqueous phase was carefully measured. The following equation was used to calculate the CSH percentage:

$$\mathrm{Hydrophobicity}\;(\%)=(1-B_{1}/B_{0})\;\times \;100$$

### 2.6. Adhesion to HT-29 Intestinal Cells

The adhesion to HT-29 cells was evaluated according to the method reported by M. Vasudha et al. [27] with slight modifications. HT-29 cells, purchased from the National Collection of Authenticated Cell Cultures (NCOACC, Shanghai, China), were cultured in MCCOY’S 5A medium (VivaCell, Shanghai, China) supplied with 10% fetal bovine serum (FBS) (Biological Industries, Kibbutz Beit-HaEmek, Israel) at 37 °C and 5% CO_2_. The experiments were performed in a six-well sterile culture plate with sterile coverslips on each well. In brief, HT-29 cells at a density of 2 × 10^5^ cells/well were added into the wells and were cultured at 37 °C and 5% CO_2_ until they reached 80% confluence. HT-29 cells were washed twice with PBS (0.1 M, pH 7.2). After culturing *L. fermentum* 664 in MRS broth for 12 h, the strains were washed with PBS twice and resuspended in FBS-free MCCOY’S 5A medium at a concentration of 1 × 10^9^ CFU/mL. For each well, 1 mL of bacterial suspension was added. After incubation for 2 h, HT-29 cells were washed five times using PBS to wash off *L. fermentum* 664 which did not adhere to HT-29 cells. Then, each well received 2 mL of methanol and stood for 10 min. Furthermore, the fixed cells were stained with Gram’s solution (at 28 °C for 5 min) and counted under a confocal microscope (Carl Zeiss, Thuringia, Germany). The adhesion rate of *L. fermentum* 664 was calculated using the following equation:

$$\mathrm{Adhesion}\;\mathrm{rate}\;(\%)=(C/C_{0})\;\times \;100$$

where *C* is the number of adherent strains, and *C*_0_ is the number of cells adhered.

### 2.7. Antimicrobial Activity

The antimicrobial activity of *L. fermentum* 664 was assessed based on the method introduced by Hassan et al. [28]. Gram-positive pathogenic bacteria (*S. aureus*) and Gram-negative pathogenic bacteria (*E. coli* and *S. enterica*) were used as indicators to investigate the antimicrobial activity of *L. fermentum* 664. The *L. fermentum* 664 was cultured in MRS broth for 12 h at 37 °C and harvested by centrifugation at 10,000 rpm for 10 min. The resulting supernatant was collected and sterilized using a sterile filter membrane with a pore size of 0.22 μm. The sterilized supernatant was then adjusted to pH 6.5. An overnight culture of pathogens was inoculated on the Luria–Bertani (LB) agar plates (Guangdong Huankai Microbiology Technology Co., Ltd., Guangzhou, China) with 6 mm diameter wells. A total of 100 μL of sterilized supernatant was poured into wells and incubated at 37 °C for 24 h, and finally, the inhibitory zone diameters were measured.

### 2.8. Bacterial Suspension Preparation

LGG, a commercially available probiotic, was used to evaluate and compare the anti-inflammatory effects of *L. fermentum* 664. LGG and *L. fermentum* 664 were separately inoculated at a concentration of 2% into MRS broth and incubated at 37 °C for 12 h. The cultures were centrifuged at 10,000 rpm for 10 min, and the bacterial cells were washed twice with PBS (0.1 M, pH 7.0). The concentration of LGG suspension was adjusted to 1 × 10^10^ CFU/mL using Roswell Park Memorial Institute (RPMI) 1640 medium. The concentration of *L. fermentum* 664 suspension was adjusted to 1 × 10^10^, 1 × 10^8^, and 1 × 10^6^ CFU/mL using RPMI 1640 medium.

### 2.9. Inflammatory Cytokines Measurement

RAW264.7 cells (purchased from the NCOACC) were cultured in the RPMI 1640 medium supplied with 10% FBS at 37 °C and 5% CO_2_. RAW264.7 cells were seeded in 48-well sterile plates (1 × 10^5^ cells/well) and incubated in a humidified incubator at 37 °C and 5% CO_2_ for 24 h. The cells were treated with or without a suspension of *LGG* or *L. fermentum* 664 for another 4 h followed by incubation with 2.5 μg/mL LPS (Sigma, St Louis, MO, USA) for an additional 12 h at 37 °C and 5% CO_2_ [29]. Cells grown in RPMI 1640 medium supplied with 10% FBS were regarded as the blank control. Thereafter, the cell supernatants were collected by centrifugation at 1000 rpm for 10 min, and contents of inflammatory cytokines including TNF-α, IL-6, and IL-1β were determined through commercial ELISA kits (BioLegend, San Diego, CA, USA) according to the manufacturer’s instructions.

### 2.10. Western Blot Analysis

RAW 264.7 cells (2 × 10^5^ cells/well) were cultivated in 6-well plates for 24 h and treated with *L. fermentum* 664 suspension or RPMI 1640 medium for 4 h. Then, LPS (2.5 μg/mL) was added to cells with incubation for 12 h. Cells grown in RPMI 1640 medium supplied with 10% FBS were regarded as the blank control. Cells were harvested, and the proteins were extracted from collected cells using a 2% sodium do-decyl sulfate (SDS) buffer. The extract was subjected to heat treatment at 100 °C for 10 min, followed by mixing with 6 × protein loading buffer. The mixture was separated using 12% SDS-PAGE gels and subsequently transferred from the gels to a polyvinylidene fluoride membrane (PVDF) (Amersham Pharmacia Biotech, Little Chalfont, UK). The membranes were blocked with 5% skim milk powder for 1 h and incubated with primary antibody against JNK (1:1000), p-JNK (1:1000), ERK (1:1000), p-ERK (1:2000), p38 (1:1000), p-p38 (1:1000), p65 (1:1000), p-p65 (1:1000), IκB-α (1:1000), p-IκB-α (1:1000), COX-2 (1:1000), internal control GAPDH (1:1000) (Cell Signaling Technology, Beverly, MA, USA), and HO-1 (1:1000) (Abcam, Cambridge, UK) at 4 °C overnight, respectively. Subsequently, they were incubated with the secondary antibody (1:5000) for 60 min at room temperature. Finally, the membranes were visualized in the dark through a luminescent image analyzer (TAITEC, Saitama, Japan).

### 2.11. Measurement of ROS

RAW264.7 cells were seeded in 96-well sterile black opaque plates at a density of 2 × 10^4^ cells per well. The plates were then placed in a humidified incubator at 37 °C and 5% CO_2_ for 24 h. The cells were then treated with or without *L. fermentum* 664 suspension for 4 h before exposure to 2.5 μg/mL LPS for 12 h [29]. Cells grown in RPMI 1640 medium supplied with 10% FBS were regarded as the blank control. The medium was removed, and the cells were washed with PBS (0.1 M, pH 7.2) twice and finally incubated with RPMI 1640 medium containing 10 μM 2’,7’-Dichlorodihydrofluorescein diacetate (DCFH-DA) (Solarbio, Beijing, China) at 37 °C for an additional 20 min in the dark. The samples were excited at 488 nm, and the fluorescence was measured at 525 nm using a fluorescence microplate reader (Thermo Scientific, Waltham, MA, USA). The following equation was used to calculate the relative ROS levels:

$$\mathrm{Relative}\;\mathrm{ROS}\;\mathrm{levels}\;(\%)=\left(C_{1}/C_{2}\right)\;\times \;100$$

where *C*_2_ is the fluorescence value of the blank control group, and *C*_1_ is the fluorescence value of the experimental groups.

### 2.12. Whole-Genome Sequencing

Genomic DNA from *L. fermentum* 664 was extracted using a DNA extraction kit (Merck, Darmstadt, Germany) and sent to Shanghai Majio Biomedical Technology Co., Ltd. (Shanghai, China) for whole genome sequencing and analysis. The genome was sequenced using a combination of Illumina sequencing platforms and Nanopore PromethION sequencing platforms. The data were utilized for bioinformatics analysis using the free online platform of Majorbio Cloud Platform (http://cloud.majorbio.com (accessed on 10 November 2022)), provided by Shanghai Majorbio Bio-pharm Technology Co., Ltd. (Shanghai, China).

### 2.13. Statistical Analysis

All experimental data were expressed as mean ± standard deviation (S.D). The SPSS software (27.0, IBM, New York, NY, USA) was used for statistical analysis. The one-way analysis of variance (one-way ANOVA) and Duncan’s multiple range test were used to determine differences between groups. All histograms were based on OriginPro software (2021, OriginLab, Northampton, MA, USA). A *p*-value under 0.05 was considered statistically significant.

## 3. Results

### 3.1. Acid Resistance and Bile Salt Tolerance

Probiotics need to go through the challenging gastrointestinal environment, characterized by low acid and high concentrations of bile salts, to arrive at the host gut microbiome. After 4 h exposure to low pH, the survival rate of *L. fermentum* 664 was 107 ± 5% (Table 1). Similarly, at a 0.3% (*w*/*v*) concentration of bile salts, *L. fermentum* 664 exhibited a survival rate of 109 ± 4% after incubation for 2 h.

### 3.2. Hydrophobicity and Adhesion Abilities

**Figure A1:**
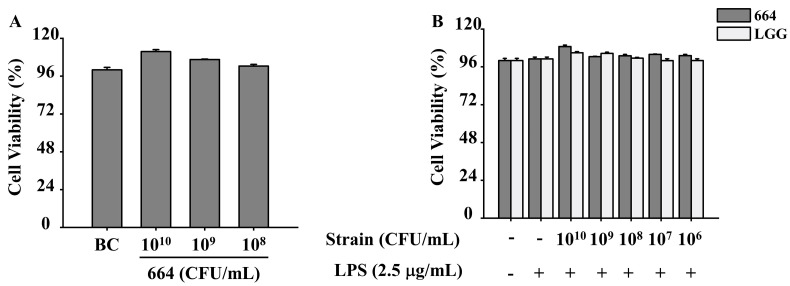
The effect of different concentrations of *L. fermentum* 664 suspension on the viability of HT-29 cells (**A**). BC: Blank control. The effect of different concentrations of *L. fermentum* 664 suspension as well as LGG suspension on the viability of RAW264.7 cells (**B**).

Cell surface hydrophobicity and adhesion to HT-29 cells are important indices to assess the adhesion potential of probiotics. Xylene, a polar solvent, is commonly used to evaluate cell surface hydrophobicity. Preliminary experiments revealed that *L. fermentum* 664 did not exhibit toxicity towards HT-29 cells (data are shown in Appendix A (Figure A1A)). As shown in Table 1, *L. fermentum* 664 exhibited 54.21 ± 5% hydrophobicity. Meanwhile, the adhesion rate to HT-29 cells was 89.53 ± 6.65%.

### 3.3. Antimicrobial Activity

The antimicrobial activity of *L. fermentum* 664 supernatant was evaluated using the agar well diffusion assay, and the results are shown in Table 2. *L. fermentum* 664 supernatant exhibited antimicrobial activities against three pathogens, but the degree of antimicrobial activity varied depending on the specific pathogen. The supernatant exhibited the strongest inhibitory effect against *S. aureus*, with an inhibition zone diameter of 20.70 ± 0.29 mm. The inhibitory effect of the supernatant against *E. coli* was weaker, with an inhibition zone diameter of 19.36 ± 0.07 mm.

### 3.4. Inhibition of Pro-Inflammatory Cytokines Release in RAW264.7 Cells by L. fermentum 664

**Figure 1:**
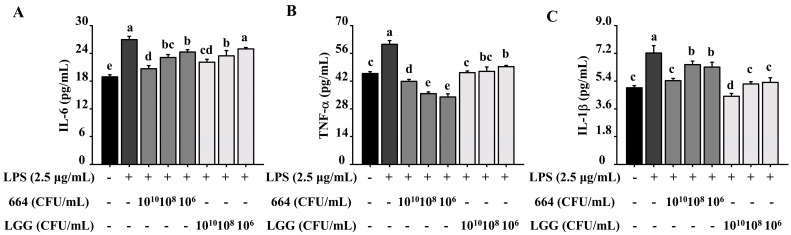
Effects of *L. fermentum* 664 or LGG treatments on the expression of immune indicators interleukin-6 (IL-6) (**A**), tumor necrosis factor-α (TNF-α) (**B**), and interleukin-1β (IL-1β) (**C**). All data are expressed as the mean ± SD (*n* = 3). Superscripts (a–e) are substantially different from one another (*p* < 0.05).

To assess the anti-inflammatory properties, RAW 264.7 cells were treated with *L. fermentum* 664 or LGG suspensions at various concentrations and subsequently stimulated with a commonly used antigen, LPS. The results of CCK-8 assays indicated that there was no observed cytotoxic effect of *L. fermentum* 664 and LGG on RAW 264.7 cells (data are shown in Appendix A (Figure A1B)). Compared to the blank control, LPS increased the release of inflammatory cytokines (IL-6, TNF-α, and IL-1β) in RAW264.7 cells. However, these cytokines were significantly inhibited when the cells were pretreated with *L. fermentum* 664 at different concentrations (1 × 10^10^, 1 × 10^8^, and 1 × 10^6^ CFU/mL) (Figure 1). When cells were treated with a concentration of 1 × 10^10^ CFU/mL of *L. fermentum* 664, the secretion levels of IL-6 and IL-1β decreased the most, with reductions of 24.0% and 24.8%, respectively. However, the most significant reduction in TNF-α secretion was observed when cells were treated with a concentration of 1 × 10^6^ CFU/mL of *L. fermentum* 664, showing a decrease of 43.9%. Compared to the LGG group, *L. fermentum* 664 exhibited a stronger inhibitory effect on the production of IL-6 and TNF-α. However, its inhibitory effect on IL-1β was inferior to that of LGG. These results suggested that *L. fermentum* 664 demonstrates promising anti-inflammatory properties.

### 3.5. Inhibition of NF-κB Activation and IκB-α Phosphorylation in RAW264.7 Cells by L. fermentum 664

**Figure 2:**
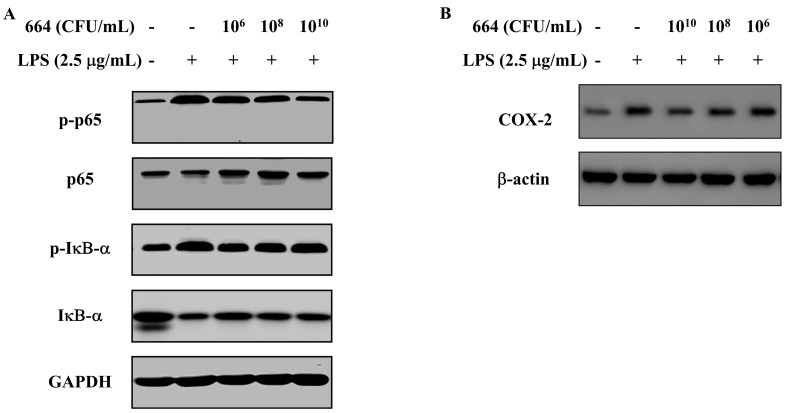
Effects of different concentrations of *L. fermentum* 664 on nuclear factor κB (NF-κB) (**A**) and cyclooxygenase-2 (COX-2) (**B**) expression in the cell model.

NF-κB plays a crucial role as a transcription factor in regulating the production of pro-inflammatory cytokines in activated macrophages. Hence, the inhibitory effect of *L. fermentum* 664 on the NF-κB signaling pathway was investigated in LPS-stimulated RAW 264.7 cells. As shown in Figure 2A, LPS enhanced the expression of phosphorylated IκB-α and p65 (p-IκB-α and p-p65) compared with the blank control. This indicated that the complex formed by NF-κB and IκB-α undergoes degradation, resulting in the activation of NF-κB protein. However, pre-treatment with *L. fermentum* 664 inhibited the phosphorylation of IκB-α and p65. These results indicated that the downregulated pro-inflammatory cytokines following treatment with *L. fermentum* 664 may be associated with blocking the NF-κB signaling pathway.

### 3.6. Suppression of COX-2 Expression by L. fermentum 664

To determine whether COX-2 is implicated in the anti-inflammatory effect of *L. fermentum* 664, the level of COX-2 expression was examined in LPS-stimulated RAW 264.7 cells pre-treated with *L. fermentum* 664. As shown in Figure 2B, treatment with *L. fermentum* 664 resulted in a concentration-dependent inhibition of COX-2 production. The downregulation of COX-2 expression by *L. fermentum* 664 may contribute to its inhibitory effects on the production of pro-inflammatory cytokines induced by LPS.

### 3.7. Inhibition of MAPK Activation in RAW264.7 Cells by L. fermentum 664

**Figure 3:**
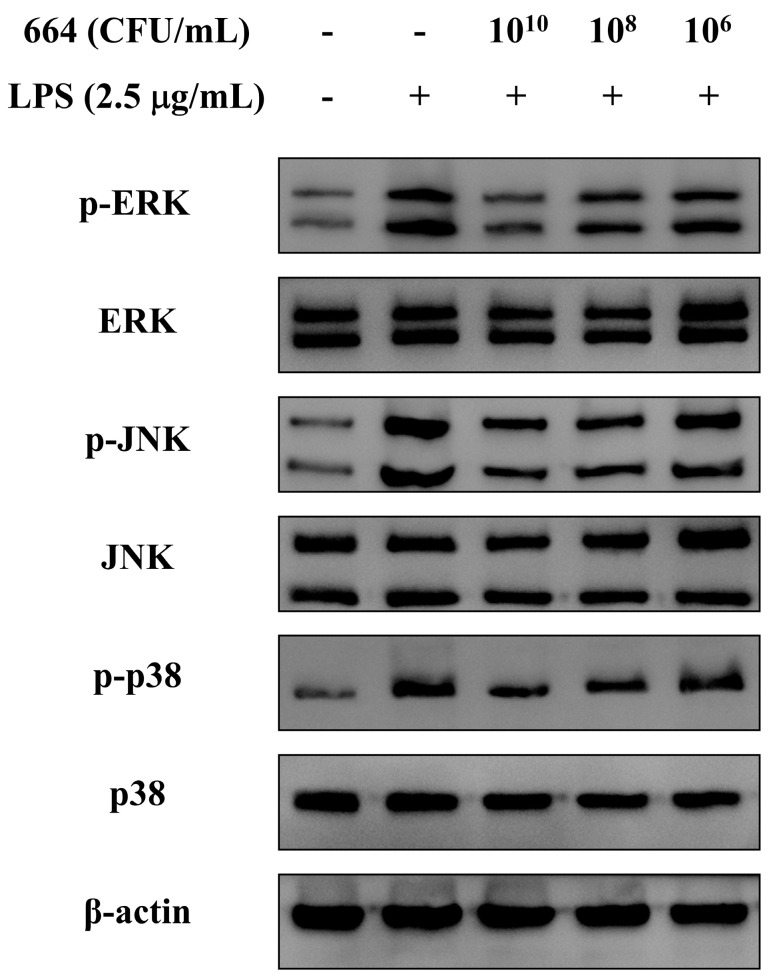
Effects of different concentrations of *L. fermentum* 664 on mitogen-activated protein kinases (MAPKs) expression in the cell model.

During the inflammatory process, MAPKs are activated as the main signaling pathway and can induce the activation of inflammatory-related proteins such as NF-κB. Therefore, we evaluated the regulatory effect of *L. fermentum* 664 on MAPKs activated by LPS. Compared to the blank control, LPS increased the phosphorylation of MAPK proteins including ERK, JNK, and p38 (Figure 3). On the contrary, the pretreatment of RAW264.7 cells with *L. fermentum* 664 can inhibit the LPS-induced phosphorylation of MAPKs in a dose-dependent manner. Moreover, the inhibitory effect is most pronounced at a bacterial suspension concentration of 1 × 10^10^ CFU/mL. The above results indicated that *L. fermentum* 664 attenuated LPS-induced inflammation by inhibiting the phosphorylation of MAPK proteins in macrophage cells.

### 3.8. Suppression of ROS and HO-1 Expression by L. fermentum 664

**Figure 4:**
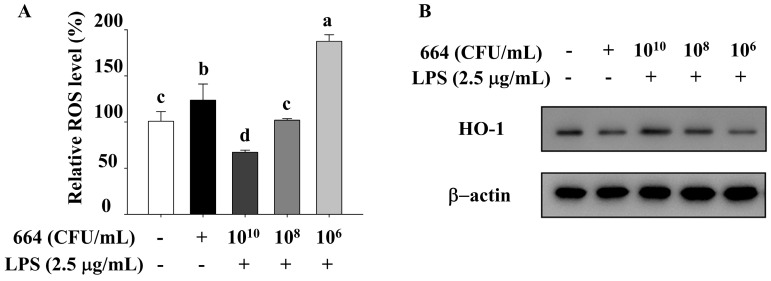
*L. fermentum* 664 regulated reactive oxygen species (ROS) and heme oxygenase-1 (HO-1) expression in LPS-stimulated RAW264.7 cells. (**A**) Effects of *L. fermentum* 664 on the relative ROS levels in lipopolysaccharide (LPS)-stimulated RAW264.7. (**B**) Effects of *L. fermentum* 664 on the expression of HO-1 protein in the cell model. Letters (a–d) are substantially different from one another (*p* < 0.05).

In redox systems in vivo, excessive ROS which cannot be eliminated promptly will exacerbate the inflammatory response. HO-1 is an antioxidant enzyme that can downregulate inflammation and ROS production. Therefore, we detected their expression in LPS-stimulated RAW264.7 cells. Compared to the LPS-stimulated cells, *L. fermentum* 664 at two concentrations (1 × 10^10^ and 1 × 10^8^ CFU/mL) significantly decreased the ROS generation while significantly increasing the expression of HO-1 protein (Figure 4). However, an increase in ROS generation and a decrease in HO-1 protein expression were observed in the group treated with *L. fermentum* 664 at a concentration of 1 × 10^6^ CFU/mL.

### 3.9. Analysis of the Genome Properties, Antibiotic Resistance and Reducing Oxidative Stress Potential of L. fermentum 664

**Figure 5:**
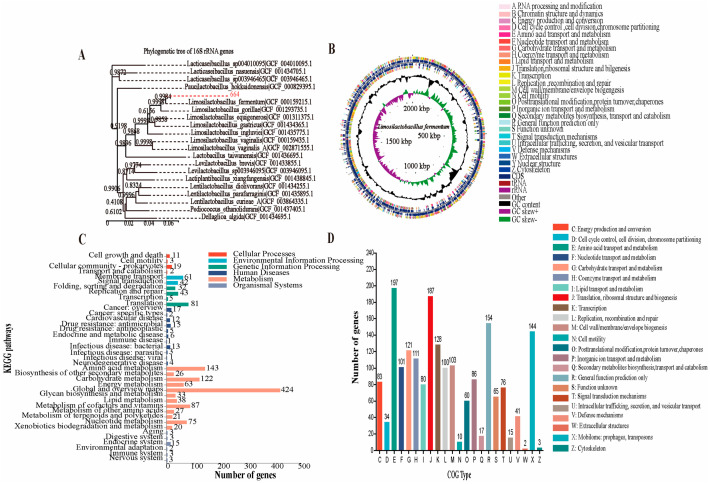
Analysis of the genome properties of *L. fermentum* 664. (**A**) The phylogenetic tree. (**B**) Genome circle. From the outside to the center: Clusters of Orthologous Groups (COG) annotation for coding deoxyribonucleic acid sequences (CDS) (colored according to COG categories) in the forward strand, CDS, transfer RNA (tRNA), and ribosomal DNA (rRNA) in the forward strand, COG annotation for CDS in the reverse strand, CDS, tRNA, and rRNA in the reverse strand, GC content (higher-than-average GC content: outward portion, lower-than-average GC content: inward portion), GC-Skew value (higher-than-average values: green, lower-than-average values: purple), and genome size. (**C**) The pathways of functional annotations for Kyoto Encyclopedia of Genes and Genomes (KEGG). The ordinate represents the Level 2 hierarchy of the KEGG pathway and the different column colors represent the Level 1 classification of the KEGG pathway. (**D**) The COG function annotation.

To obtain functional information, the whole genome sequencing of *L. fermentum* 664 was analyzed. The results of a DNA and National Center for Biotechnology Information (NCBI) gene pool comparison showed that strain 664 had 99.93% homology with *L. fermentum*. As a result, strain 664 was identified as *L. fermentum*, and its phylogenetic tree was shown in Figure 5A. The genomic circle of *L. fermentum* 664 was shown in Figure 5B. The genome sequences of *L. fermentum* 664 consisted of approximately 2,060,326 base pairs with a GC content of 51.72%. It contained 2006 coding deoxyribonucleic acid sequences (CDS), accounting for 85.7% of the total genes. Additionally, there were 15 ribosomal DNA (rRNA) genes and 58 transfer RNA (tRNA) genes present in the genome (Table 3). To detect antimicrobial resistance genes, the assembled sequences were compared with the reference sequences in the ResFinder database (https://cge.cbs.dtu.dk/services/ResFinder/ (accessed on 25 November 2022)) using ResFinder_v4.1.0. No antibiotic resistance genes were detected in *L. fermentum* 664. In addition, we investigated the presence of mobile genetic elements including plasmids and transposons. The results demonstrated that *L. fermentum* 664 possesses 23 transposons, while no plasmids were observed. Figure 5C shows the distribution of functional annotations for the Kyoto Encyclopedia of Genes and Genomes (KEGG) database of *L. fermentum* 664. A total of 1486 genes of *L. fermentum* 664 were annotated in the KEGG database, which were annotated to six major functions and 39 pathways. The genome of *L. fermentum* 664 was mainly annotated for Metabolism (1079), Genetic Information Processing (161), Environmental Information Processing (103), Human Diseases (79), Cellular Processes (35), and Organismal Systems (29) pathways. Table 4 shows the genes associated with regulating oxidative stress in KEGG annotation. The function annotation of the Clusters of Orthologous Groups (COG) of *L. fermentum* 664 was shown in Figure 5D. The main COG categories were Amino acid transport and metabolism (197), Translation, ribosomal structure and biogenesis (187), General function prediction only (154), Mobilome: Among the features of prophages, transposons (144), and Transcription (128). The number of genes with Unknown function was 65.

## 4. Discussion

Traditional drugs for inflammation, such as non-steroidal anti-inflammatory drugs (NSAIDs), have various side effects [30]. In recent decades, lactobacillus and traditional fermented foods have been extensively reported to prevent and treat gastrointestinal illnesses and inflammation owing to their good biological efficacy and safety. LAB is also advocated as a novel anti-inflammatory medicine [31,32,33]. Deni et al. [7] indicated that *L. plantarum* 1K can produce secondary metabolites with strong anti-inflammatory properties, suggesting its potential to be used as an anti-inflammatory agent. However, the mechanism by which this occurs was not studied in depth. In the present study, we identified that *L. fermentum* 664, a novel probiotic strain isolated from Chinese traditional fermented pickles, has anti-inflammatory properties. Moreover, the mechanism of the anti-inflammatory effect was also elucidated.

Upon incorporation into the daily diet, live probiotics face various antimicrobial factors in the gastrointestinal tract such as low pH and bile salts at high concentrations [34]. The pH in the stomach is typically between 2.0 and 3.5, and food remains there for approximately 2 to 4 h. In this study, *L. fermentum* 664 exhibited exceptional survival rates at pH 2.0, reaching up to 107 ± 5%. The acid tolerance of *L. fermentum* 664 may be related to the presence of H-ATPases in the cells. This enzyme can transfer excessive protons from the intracellular space to the extracellular environment, thereby maintaining the pH homeostasis within the cell [35]. The intestine serves as another barrier for probiotics to enter the human body, containing bile salts at concentrations of 0.15% to 0.3%. Bile salts can induce protein denaturation on the cell membrane, leading to the disruption of cell integrity. Additionally, bile salts can also chelate with intracellular metal ions, causing oxidative damage to DNA [36]. In this study, the survival rate of *L. fermentum* 664 cultured in an environment with 0.3% bile salts for 2 h is 109 ± 4%. This may be attributed to the production of bile salt hydrolase enzymes or alterations in the composition of the cell membrane by *L. fermentum* 664 [9].

Probiotics with high adhesive ability can continuously exert their functional characteristics in the body and, through competition with pathogens, prevent their colonization in the gastrointestinal tract [37]. The bacterial adhesion mechanism is a complex process that involves both nonspecific interactions, such as hydrophobic interactions and electrostatic forces, as well as specific interactions, such as receptor–protein interactions. Therefore, in this study, the adhesiveness of *L. fermentum* 664 was evaluated by assessing its hydrophobicity and its ability to adhere to HT-29 cells [27,38]. In the present study, *L. fermentum* 664 exhibited a hydrophobicity level of 54.21 ± 5%. High hydrophobicity allows increased interaction between probiotics and host epithelial cells [34]. The adherence ratio of *L. fermentum* 664 to HT-29 cells, a kind of human intestinal epithelial cell, was 89.53 ± 6.65%. Similar to our work, the adhesion ratios of *L. fermentum* sp. GV254 [27] and *Lacticaseibacillus paracasei* RAMULAB16 [23] were 81.14% and 88.53%, respectively. The results above suggested that *L. fermentum* 664 exhibited the potential to colonize the gastrointestinal tract to exert its probiotic effects.

Antimicrobial activity is one of the important functions of probiotics, and it helps maintain the stability of the gut microbiota. Probiotics can inhibit the proliferation of pathogenic microorganisms in the host’s body through multiple mechanisms: (1) production of antimicrobial substances; (2) competition for nutrients or adherence sites with pathogenic bacteria; and (3) regulation of intestinal barrier function and the immune system [39]. In this study, we discovered that the fermentation supernatant of *L. fermentum* 664 exhibited inhibitory effects on three common pathogenic bacteria, including Gram-positive bacteria (*S. aureus*) and Gram-negative bacteria (*E. coli* and *S. enterica*). The *L. fermentum* isolated from Tulum cheese showed smaller inhibition zones against *E. coli* and *S. aureus* compared to *L. fermentum* 664 [40], indicating that *L. fermentum* 664 possesses better antimicrobial activity. Additionally, we observed that the fermentation supernatant of *L. fermentum* 664 retained its inhibitory effect on the three pathogenic bacteria even after adjusting the pH to 6.5. Therefore, the antimicrobial activity of *L. fermentum* 664 may be attributed to secreted antimicrobial substances, such as antimicrobial peptides [41].

LPS, a molecule found in the cell wall of bacteria, is commonly recognized as an inducer of inflammation in the monocyte/macrophage cell lineage. It can bind to LPS receptors on target cells, such as the Toll-like receptor 4 (TLR4), triggering the expression of inflammatory cytokines and inflammatory proteins, ultimately leading to the occurrence of inflammation [42]. Pro-inflammatory cytokines can alter the permeability of the vascular endothelium or recruit neutrophils and excessive plasma to the site of infection, thereby amplifying the inflammation. In addition, excessive pro-inflammatory cytokines can also stimulate the production of prostaglandins (PGs), leading to symptoms such as pain and fever in the host [43,44]. Thus, modulating the production of pro-inflammatory cytokines is an effective strategy for mitigating inflammation. Compared to the positive control (LGG), *L. fermentum* 664 exhibited a better inhibition of the production of TNF-α and IL-6 in LPS-stimulated RAW264.7 cells. However, the optimal concentration of *L. fermentum* 664 to inhibit the release of distinct inflammatory cytokines was different. These results were consistent with a previous study [45]. We speculated that the regulation of the expression of different pro-inflammatory cytokines may be attributed to multiple signaling pathways.

NF-κB is one of the most essential signaling pathways in immune response and inflammation. It is composed of a dimer formed by p65 and p50 subunits and is responsible for the expression of inflammatory mediators and cytokines [46,47]. In resting cells, NF-κB, which associates with IκB-α, is present in the cytoplasm as an inactive heterodimer. When cells are stimulated by inflammatory inducers such as LPS and ROS, the activation of IκB kinase (IKK) leads to the phosphorylation and degradation of IκB-α, resulting in the protein activation of NF-κB. Subsequently, NF-κB is translocated from the cytoplasm to the cell nucleus, selectively inducing the gene expression of pro-inflammatory mediators such as TNF-α and inducible nitric oxide synthase (iNOS) [48]. In our study, *L. fermentum* 664 was found to significantly inhibit the LPS-induced activation of NF-κB by decreasing the phosphorylation of IκB-α and p65 in RAW264.7 cells. COX-2 is commonly regarded as a downstream modulator of NF-κB. COX-2 is capable of converting arachidonic acid into PGs, specifically prostaglandin E2 (PGE2). Excessive PGE2 exacerbates the inflammatory response by increasing vascular permeability and stimulating the production of pro-inflammatory mediators such as IL-6 and chemokines [30,49]. Selective COX-2 inhibitors have recently emerged as promising treatments for inflammation, providing effective therapeutic options with minimized side effects [30]. In the present study, *L. fermentum* 664 significantly reduced the production of COX-2 induced by LPS in a concentration-dependent manner. Many studies have demonstrated that probiotics can exert anti-inflammatory effects by blocking the activation of the NF-κB signaling pathway and suppressing the expression of COX-2. In 2,4,6-trinitrobenzene sulfonic acid-induced colitis mice, *L. fermentum* IM12 downregulated the release of inflammatory cytokines by suppressing the expression of iNOS and COX-2. Additionally, it inhibited the activation of NF-κB and signal transducer and activator of transcription 3 (STAT3) [50]. *Lactobacillus casei* 3260 had been shown to decrease the production of pro-inflammatory mediators, including COX-2 and TNF-α by blocking the activation of NF-κB in LPS-induced RAW264.7 cells [51]. Therefore, we speculated that *L. fermentum* 664 exerted an anti-inflammatory effect by suppressing NF-κB activation and COX-2 expression.

The activation of MAPK signaling pathways promotes the inflammatory response by regulating the expression of inflammatory mediators (TNF-α, IL-6, and NO) and signaling pathways (NF-κB) [52]. ERK, JNK, and p38 are the major proteins in the MAPK family, which are activated by different subtypes of MAPK kinases. After activation, ERK can regulate the expression of inflammatory genes by participating in the modulation of cellular processes such as cell differentiation, meiotic division, and post-mitotic functions. The activation of JNK and p38 can activate the IKK protein, thereby inducing the expression of NF-κB protein [53]. In this study, *L. fermentum* 664 was found to reduce the phosphorylation of ERK, JNK, and p38 proteins induced by LPS in a concentration-dependent manner. Similar to our study, Sakshi et al. [54] reported that the inhibition of pro-inflammatory mediator production in LPS-induced RAW264.7 cells by *L. fermentum* V may be attributed to the suppression of the MAPK pathway activation that in turn regulated NF-κB signaling. These results suggested that *L. fermentum* 664 can inhibit the activation of the NF-κB pathway by blocking the MAPK signaling pathway. Research studies have discovered the activation of MAPK kinases in relation to the upregulation of ROS expression during inflammation [55]. Consequently, we examined the effect of *L. fermentum* 664 on ROS levels in LPS-stimulated RAW264.7 cells.

The production of ROS during the inflammatory process is inevitable for the host’s immune response to eliminate pathogens. When macrophages and neutrophils arrive at the site of inflammation, they phagocytize pathogens and dead cells. This process activates nicotinamide adenine dinucleotide phosphate (NADPH) oxidase on the cell membrane. The NADPH oxidase converts oxygen into ROS, which is released into the intracellular or peripheral tissues to inhibit the activity of pathogens [56]. If the endogenous antioxidant system fails to promptly eliminate excessive ROS within the body, ROS can induce oxidative stress. Oxidative stress can activate the NF-κB and MAPK signaling pathways and exacerbate systemic inflammation, thus establishing a detrimental cycle [57,58]. However, ROS detoxification and redox homeostasis can be promoted through the expression of antioxidation enzymes [59]. HO-1, a well-recognized cellular protective enzyme, can break down toxic heme and generate antioxidants. In addition, HO-1 can induce the production of the anti-inflammatory cytokine IL-10 to alleviate inflammation [60]. The present study demonstrated that *L. fermentum* 664 reduced ROS content and increased HO-1 protein expression in LPS-stimulated RAW264.7 cells. When the bacterial concentration was at the highest, the best moderating effect was reached. High levels of HO-1 expression can inhibit the production of ROS. Similar to our findings, Binbin et al. [37] also found that the culture supernatants of *Lactobacillus gasseri* (*L. gasseri*) FWJL-4 and *L. plantarum* Fjias-5 both suppressed ROS and pro-inflammatory cytokine production in RAW264.7 cells, which were associated with the enhanced expression levels of HO-1. Therefore, *L. fermentum* 664 may alleviate oxidative stress to inhibit inflammation in LPS-stimulated RAW264.7 cells, potentially through its metabolic products. However, when the concentration of *L. fermentum* 664 suspensions reached 1 × 10^6^ CFU/mL, the relative ROS level in LPS-stimulated RAW264. 7 cells was promoted. In addition, the expression of HO-1 protein was also suppressed. In our previous study, *L. fermentum* 664 at a concentration of 1 × 10^6^ CFU/mL was found to inhibit the production of inflammatory cytokines induced by LPS. The above phenomenon may be due to the fact that *L. fermentum* 664, at a concentration of 1 × 10^6^ CFU/mL, may produce both anti-inflammatory substances and substances that induce the rise of ROS. Bacteria can produce different metabolic products to adapt to the environment at different concentrations. This indicates that the anti-inflammatory mechanism of *L. fermentum* 664 suspensions varies at different concentrations. We are interested in exploring the mechanisms behind this phenomenon and plan to conduct further research in the future.

By utilizing high-throughput technology to analyze the complete genome sequence, one can rapidly obtain fundamental information about a target strain. Gene annotation allows for the rapid acquisition of relevant functional genes and safety information [61]. Thus, a whole genome sequencing of *L. fermentum* 664 was performed to evaluate its safety and anti-inflammatory potential based on genes and genomes. Antibiotics are commonly used to kill invasive pathogens or limit their replication to control the development of infection within the host. Antibiotic resistance ensures the viability of a probiotic within the host. However, if these antibiotic resistance genes are transferred to pathogenic bacteria in the gastrointestinal tract, it can render antibiotics ineffective. It has been established that antibiotic resistance genes can be transferred through three mechanisms: conjugation, transformation, and transduction. Conjugation is the primary mode of transfer for antibiotic resistance genes. Antibiotic resistance genes can be transferred through their association with plasmids and transposons [62,63]. In the present study, no antibiotic resistance genes were detected in *L. fermentum* 664. Investigating the transferability of antibiotic resistance genes did not necessitate the study of transposons and plasmids in *L. fermentum* 664. Therefore, *L. fermentum* 664 is deemed safe in terms of antibiotic resistance. KEGG is a database for genes and genome biological interpretation [64]. By comparison with the genes and genomes annotated in KEGG, we found that *L. fermentum* 664 had functional genes associated with antioxidant and anti-inflammatory proteins including Cytochrome bd ubiquinol oxidase subunit I (CydA), Cytochrome bd ubiquinol oxidase subunit II (CydB), and NAD(P)H dehydrogenase quinone 1 (NQO1). Additionally, CydA and CydB can indirectly regulate cellular redox homeostasis by encoding cytochrome bd-type oxidases [65]. Some studies have shown that NQO1, acting as an endogenous antioxidant, has a mitigating effect on inflammation [66]. It can be concluded that *L. fermentum* 664 had the potential to inhibit inflammation by regulating oxidative stress. The COG database was developed to facilitate the phylogenetic classification of proteins derived from complete microbial genomes [67]. In COG analysis, the majority of protein sequences from *L. fermentum* 664 genomes were assigned to the functional categories of Amino acid transport and metabolism, as well as Translation and ribosomal structure and biogenesis. Increasing evidence has reported that probiotics can inhibit inflammation and oxidative stress by secreting polypeptide proteins and polysaccharides [68,69]. In our study, *L. fermentum* 664 exhibited anti-inflammatory effects, which might be attributed to its antioxidative capacity and secondary metabolites. However, this needs to be further verified.

## 5. Conclusions

In conclusion, we have demonstrated that *L. fermentum* 664 isolated from Chinese fermented pickles has the potential to become a probiotic with anti-inflammatory properties. The strain exhibited acid and bile salt tolerance, adhesion capability, antimicrobial activity, and acceptable safety profile. Furthermore, *L. fermentum* 664 reduced the expression of inflammatory cytokines and COX-2 protein by blocking the activation of MAPK and NF-κB signaling pathways induced by LPS. This may be attributed to its suppression of the increase in ROS levels by upregulating the expression of HO-1 in LPS-stimulated RAW264.7 cells. Additionally, the anti-inflammatory capacity of *L. fermentum* 664 may be associated with its antioxidative ability and secondary metabolites, as suggested by whole genome sequencing. However, further experiments are needed to explore this in detail. Thus, *L. fermentum* 664 can be considered a candidate for preventing oxidative stress and inflammation-associated disorders.

## Figures and Tables

**Table 1:** Probiotic properties of *Limosilactobacillus fermentum* 664.

Characteristics	Rate (%) *
Acid resistance	107 ± 5
Bile salt tolerance	109 ± 4
Hydrophobicity	54.21 ± 5
Adhesion	89.53 ± 6.65

*: Data are presented as mean ± standard deviation.

**Table 2:** Antimicrobial activities of *L. fermentum 664* supernatants against pathogens.

Pathogen	Inhibitory Zone Diameter (mm) *
*S. aureus*	20.70 ± 0.29 ^a^
*E. coli*	19.36 ± 0.07 ^c^
*S. enterica*	20.27 ± 0.01 ^b^

*: Letters (a–c) are substantially different from one another (*p* < 0.05).

**Table 3:** Genomic characteristics of *L. fermentum* 664.

Genomic Characteristics	Explanation
Identification	*Limosilactobacillus fermentum*
Genome size (bp)	2,060,326
GC contents (%)	51.72
CDS	2006
CDS/total genes (%)	85.7
No. of rRNA genes	15
No. of tRNA genes	58

**Table 4:** KEGG annotated genes that regulate oxidative stress.

Gene ID	KO ID	KO Name	KO Description
gene0309	K00425	cydA	cytochrome bd ubiquinol oxidase subunit I
gene0310	K00426	cydB	cytochrome bd ubiquinol oxidase subunit II
gene0759	K00355	NQO1	NAD(P)H dehydrogenase quinone 1

## Data Availability

The data are contained within this article.

## Appendix A

1. HT-29 cells were treated or untreated with *L. fermentum* 664 suspensions of varying concentrations for 2 h, followed by assessing cell viability using the Cell Counting Kit-8 (CCK-8) assay. The results indicated that the viability of HT-29 cells was not inhibited by *L. fermentum* 664 (Figure A1A).

2. RAW264.7 cells were treated or untreated with suspensions of LGG or *L. fermentum* 664 at varying concentration for 4 h, followed by incubation with 2.5 μg/mL of LPS. Cell viability was assessed using the CCK-8 assay. The results indicated that the viability of RAW264.7 cells was not inhibited (Figure A1B).
